# Comparing the effectiveness of asynchronous e-modules and didactic lectures to teach electrocardiogram interpretation to first year US medical students

**DOI:** 10.1186/s12909-023-04338-6

**Published:** 2023-05-22

**Authors:** Doreen M. Olvet, Kaveh Sadigh

**Affiliations:** 1 Department of Science Education, Donald and Barbara Zucker School of Medicine at Hofstra/Northwell, Hempstead, NY 11549 USA; 2 Department of Medicine, Renaissance School of Medicine, Stony Brook University, Stony Brook, New York, 11794 USA

**Keywords:** Electrocardiogram, e-module, Didactic lecture, Preclinical, Medical students, Residents

## Abstract

**Background:**

Medical students are expected to be competent in interpreting electrocardiograms (ECGs) by the time they graduate, but many are unable to master this skill. Studies suggest that e-modules are an effective way to teach ECG interpretation, however they are typically evaluated for use during clinical clerkships. We sought to determine if an e-module could replace a didactic lecture to teach ECG interpretation during a preclinical cardiology course.

**Methods:**

We developed an asynchronous, interactive e-module that consisted of narrated videos, pop-up questions and quizzes with feedback. Participants were first year medical students who were either taught ECG interpretation during a 2-hour didactic lecture (control group) or were given unlimited access to the e-module (e-module group). First-year internal medicine residents (PGY1 group) were included to benchmark where ECG interpretation skills should be at graduation. At three time-points (pre-course, post-course, and 1-year follow-up), participants were evaluated for ECG knowledge and confidence. A mixed-ANOVA was used to compare groups over time. Students were also asked to describe what additional resources they used to learn ECG interpretation throughout the study.

**Results:**

Data was available for 73 (54%) students in the control group, 112 (81%) in the e-module group and 47 (71%) in the PGY1 group. Pre-course scores did not differ between the control and e-module groups (39% vs. 38%, respectively). However, the e-module group performed significantly better than the control group on the post-course test (78% vs. 66%). In a subsample with 1-year follow-up data, the e-module group’s performance decreased, and the control group remained the same. The PGY1 groups’ knowledge scores were stable over time. Confidence in both medical student groups increased by the end of the course, however only pre-course knowledge and confidence were significantly correlated. Most students relied on textbooks and course materials for learning ECG, however online resources were also utilized.

**Conclusions:**

An asynchronous, interactive e-module was more effective than a didactic lecture for teaching ECG interpretation, however continued practice is needed regardless of how students learn to interpret ECGs. Various ECG resources are available to students to support their self-regulated learning.

**Supplementary Information:**

The online version contains supplementary material available at 10.1186/s12909-023-04338-6.

## Introduction

Electrocardiogram (ECG) interpretation is an essential skill used clinically to evaluate and diagnose patients with active cardiac disease such as arrythmias and acute coronary syndrome [1]. Medical students are expected to be competent in ECG interpretation before graduating [2]. However there seems to be a gap between the expected level of competence and medical students’ ability to interpret ECGs [3–6]. This disparity also extends to residency and beyond [7–11]. Medical students don’t feel confident about their ECG interpretation skills and have expressed their frustration with learning this skill [12, 13].

Teaching ECG interpretation has been described in the literature as challenging [6, 14]. Didactic lectures and teaching rounds are most often used to teach students how to interpret ECGs [15] but other methods have been investigated, including concept maps [16], deliberate practice [17], puzzle-based methods [18], and near-peer teaching [19], all with mixed results. ECG interpretation is a technical skill that needs to be practiced often to master [20, 21], therefore some level of self-directed learning (SDL) and practice is needed to maintain skill level. However, several studies that used SDL as the primary pedagogical method for teaching ECG interpretation have failed to show that it was a good method [22, 23].

Web-based learning, or e-learning, is a valuable tool which affords students flexibility in what, when and how long they interact with the material [24, 25]. Most medical students are Generation Z or Millennial learners, who tend to be more comfortable with learning material through videos and internet-based resources [26, 27] making e-learning a viable option as an educational platform. Medical students already use various online tools for learning [28, 29]. The most common format for delivering web-based ECG material is through tutorials, which include the use of text, images, videos or animations [30]. These online tutorials are typically successful at improving medical students’ ability to interpret ECGs in the short-term [13, 31–33] but not always [34, 35]. Other educators simply make their teaching materials available on an online course management system for students to review independently, which has had mixed results [36, 37]. Studies that have looked at longer retention of learning using online tutorials often show that students lose some or all of their educational gains [13, 31, 34].

E-learning resources can provide students with the opportunity for consistent, deliberate practice, which is especially relevant for learning and retaining the ability to interpret ECGs [38]. The literature on the use of e-learning resources for ECG interpretation have largely focused on medical students during their clinical phase of the curriculum, which limits the amount of time students can practice this skill throughout their time in medical school. Only one other study has looked at the use of an e-learning tool to teach ECG interpretation during a brief session in the second year of medical school but did not find a benefit when compared to near-peer teaching [39]. We developed an asynchronous, interactive e-module to facilitate ECG learning in first-year medical students (MS1s) throughout the three-week preclinical cardiology block. The objective of this study was to determine if MS1s who learned how to interpret an ECG using an e-module during their preclinical cardiology course would perform better on a knowledge exam and have more confidence than students who learned the same material in a didactic lecture. We also tested the retention of ECG interpretation skills by assessing students one year later. We included first-year internal medicine residents (PGY1s) to serve as a benchmark for where graduating medical students’ ECG interpretation skills should be.

## Methods

### Clinical context

In 2014, the Renaissance School of Medicine at Stony Brook University (RSOM) implemented the Learning focused, Experiential, Adaptive, Rigorous and Novel (LEARN) curriculum which focused on active learning and developing physician competencies in an integrated and contextual manner [40]. To allow for early exposure to clinical experiences, the foundational basic science curriculum (Phase I) was reduced to 18 months. The primary clinical phase (clerkships, Phase II) remained 12 months long and the advanced clinical phase (Phase III) was extended to 16 months.

The Cardio-Pulmonary-Renal (CPR) course is scheduled in the middle of Phase I (during March/April of the first year of the curriculum) and focuses on the physiology and pathophysiology of the cardiac, pulmonary and renal systems. The CPR course is 9 weeks long, evenly split between the three segments (i.e., 3 weeks each). The cardiology segment is heavily focused on the flipped classroom model, having the students perform required reading prior to class, and then have them apply that knowledge to unfamiliar clinical scenarios in lecture.

The ECG interpretation session took place in the first week of the cardiology segment after several days of didactic lectures on membrane potentials, histology, and the electrophysiology of the heart. Subsequent sessions topics that week included the cardiac cycle, hemodynamics, regulation and the pathophysiology of the heart. For the remainder of the course, ECG interpretation was incorporated into most case-based learning that occurred during large and small group sessions to reinforce the foundational knowledge learned in the initial session. Both the control and e-module groups participated in these case-based learning sessions.

### Participants

All MS1s in 2017 (N = 135) and 2018 (N = 139) were invited to participate in the study. In 2017, MS1s received the standard ECG curriculum (control group). In 2018, in lieu of the standard curriculum MS1s were required to use an online asynchronous module to learn ECG interpretation (e-module group). At the beginning of the cardiology course, an email was sent to all MS1s asking them to participate in the study and including a link to the ECG survey. A medical student working on the project also announced the study at the beginning of a cardiology course lecture as a follow-up to the email.

Two cohorts of first year internal medicine residents (PGY1s, resident group, N = 66) were also invited to participate in the study as a benchmark for where graduating medical students’ ECG interpretation skills should be. One of the authors (DMO) attended the residents’ weekly noon conference to recruit PGY1s to participate in the study, along with sending an email with a link to the survey. PGY1s did not have access to the e-module.

### Standard ECG curriculum (control group)

The standard ECG curriculum was delivered in a flipped-classroom model. Prior to attending the didactic session, students were expected to read a chapter on the electrocardiogram in the assigned textbook (Lilly’s “Pathophysiology of Heart Disease”). Then, students participated in a 2-hour didactic session about the 12 lead ECG, which took place in the first week of the cardiology course. The content of the session included: checking voltage calibration, interpreting the rhythm, calculating heart rate, measuring intervals (PR, QRS, QT), interpreting mean QRS axis, abnormalities of the P wave, abnormalities of the QRS (hypertrophy, bundle branch block, infarction) and abnormalities of the ST segment and T wave, which were covered in the required reading. ECG examples were shown to illustrate normal and to contrast it with abnormal ECGs, relying on student responses and interpretation of the ECGs and the thought process that led them to that interpretation.

### E-module ECG curriculum (e-module group)

An electronic ECG module was developed after the implementation of the new LEARN curriculum. In conjunction with the curriculum reform (i.e., reducing the preclinical curriculum to 18 months), there was a push to move as many resources as possible to an electronic format. To facilitate e-learning, all incoming students were given iPads to be used as their primary electronic device and faculty were encouraged to shift curriculum content to an electronic, asynchronous format.

To ensure a student perspective, three third year medical students (MS3s) worked with the cardiology course director (KS) to create the e-module structure (see screenshots in the **Supplemental Material Appendix** 1). Content from the original didactic session on ECG interpretation was used as the basis for the e-module, which was divided into six modules (Table 1). The visual aspect of the module was created using a standard slide deck. Animated handwritten text was used to pace the material. In an effort to mimic a recording of a live session, audio recordings narrated the visual content. However, unlike a live session, students were able to increase or decrease the speed of the module based on their learning style, closed captioning was made available as an accessibility feature, and students were permitted to watch each module multiple times. Student engagement and understanding of content was promoted by embedding periodic questions related to content just covered. These questions were followed by detailed explanations, after which the video resumed. At the end of each module there was a quiz to reinforce key concepts and apply what was learned to clinical scenarios. An ECG cheat sheet was available for reference with definitions of key terms (e.g., the PR interval and its normal duration). For questions that required direct analysis of an ECG image, calipers were available to assist with calculating the duration of the interval. In addition to watching the modules multiple times, students could engage the e-modules in any order, partially complete a module, or choose not to participate in the pop-up questions or quizzes, based on their own self-assessment of what material they needed to cover. Results from the questions and quizzes were not recorded. Students had unlimited access to the e-module after completing the pre-course survey.

**Table 1:** Topics covered in each of the six ECG e-modules

Module	Topics	End of module questions
Module 1: ECG basics	The normal ECG and how to interpret the ECG strip	22-question quiz
Module 2: ECG abnormalities	Atrial enlargement, ventricular hypertrophy, bundle branch blocks, pathologic Q waves and T-wave inversion	5-question quiz
Module 3: ECG Rhythms and arrhythmias	Sinus tachycardia, sinus bradycardia, atrial fibrillation, atrial flutter, Wolff-Parkinson-White syndrome, and paroxysmal supraventricular tachycardia	10-question quiz
Module 4: Atrioventricular blocks	1st degree, 2nd degree (Type 1 and Type 2), 3rd degree	6-question quiz
Module 5: Ventricular arrhythmias	Premature ventricular contractions (PVC’s), ventricular tachycardia (VT), Torsades de Pointes, ventricular fibrillation (VFib)	5-question quiz
Module 6: Ischemia/ACS	ECG signs of subendocardial ischemia as well as the acute coronary syndromes (including ST elevation MI with a focus on localizing lesions to a coronary artery)	10-question quiz

### Evaluations

A survey was distributed through Qualtrics (https://www.qualtrics.com/) to all MS1s at the beginning of the cardiology portion of the CPR course (pre-course), at the end of the CPR course approximately 3 weeks later (post-course), and 1 year later (1 year follow-up). The same survey was distributed to the PGY1s on the same timeline (starting in July), but they did not receive any ECG related educational materials. Each survey was comprised of a knowledge test and confidence ratings to examine the trainee’s ability to and confidence in identifying key aspects of ECG interpretation that were covered in the didactic and e-module sessions. For the knowledge test, respondents were shown 6 ECG rhythms and were asked to select the correct ECG interpretation from 4 choices. The pre-test and the post-test were exactly the same and the answers were not divulged to the students or trainees. The total number of correct responses was added up and a total percent correct score was calculated. For the confidence ratings, the survey prompted respondents to determine what degree to which they disagreed or agreed with each of eight statements based on a 5-point Likert scale: 1 = Strongly Disagree, 2 = Disagree, 3 = Neither Agree nor Disagree, 4 = Agree, and 5 = Strongly Agree. Students also had the option to select “not sure” for each of the statements. The eight statements all began with the statement “I feel confident in being able to identify…” and the following aspects of ECG interpretation were included as separate ratings: (1) P waves, (2) QRS waves, (3) T waves, (4) acute myocardial infarction, (5) the area affected by the ischemia, (6) the source of a lesion, (7) arrhythmias (atrial fibrillation, atrial flutter, ventricular tachycardia, ventricular fibrillation), and (8) heart blocks (first degree, second degree, third degree). A total confidence score was calculated by summing the eight Likert scale responses (for a maximum score of 40).

All students were asked if they used additional resources to learn ECGs prior to the survey. There was an open-ended text box available for students to describe what additional resources they used. For the e-module group, students were asked to report how much of each module they reviewed on the following scale: none, some, or all.

### Statistical analysis

Data were statistically evaluated using IBM SPSS Statistics (SPSS Inc., Chicago, Illinois, USA, Version 22.0). Descriptive statistics are presented as the mean (and standard deviation) for the knowledge percent score and total confidence scores. The knowledge percent score was analyzed using a mixed analysis of variance (ANOVA) to determine if there was an interaction between group (control, e-module and PGY1) and time (pre-course, post-course and 1 year follow-up). Post-hoc tests were used to examine specific interaction effects, either between (Student’s *t*-test) or within-groups (paired *t*-tests). The total confidence score was negatively skewed at the post-course and 1-year follow-up time-points, therefore nonparametric tests were used to examine differences between and within groups. The Mann-Whitney U test (U statistic) was used to compare differences between groups at each time-point. The Wilcoxon Signed Rank test (Z statistic) was used to determine differences over time (pre- vs. post-course and post-course vs. 1-year follow-up). The relationship between knowledge and confidence scores was determined using Spearman’s rank correlation coefficient (*r*_*s*_*)*. To correct for family-wise error within each outcome, Bonferroni correction was applied and an adjusted (adj) *p*-values are presented when applicable. Chi-square tests (*χ*^*2*^) were used to determine differences between the control and e-module groups on the types of resources that they used to learn ECG interpretation. A *p* value ≤ 0.05 was considered statistically significant. This study was deemed exempt from review by the Stony Brook University Institutional Review Board (Protocol #1026352). Informed consent was obtained from all subjects.

## Results

### Participants

In the control group, 73 (out of 135, 54%) had a complete set of pre- and post-course data. In the e-module group, a complete set of pre- and post-test data was obtained for 119 students, however 7 of those students reporting not using the e-module during the course so they were removed from the analysis, leaving 112 (out of 139, 81%) students. In the PGY1 group, 47 residents (out of 66, 71%) had complete data.

To determine if there was a sampling bias, we compared pre-course knowledge scores of students who were included in the study and those who were not included (i.e., those with pre-course data only) within each group. Participants who did not complete a post-course survey had a significantly lower pre-test knowledge score compared to those who were included (control: mean = 31.9%, SD = 15.1%; *t*(107)=-1.9, *p* = 0.03; e-module: mean = 30%, SD = 17.3%; *t*(135)=-1.8, *p* = 0.04). Mean knowledge score for the PGY1 trainees who were not included in the study was not significantly different than those who were (*p* = 0.09). There was no significant difference in confidence score when comparing participants who were and were not included for all three groups (all *p*-values > 0.05).

### E-module use

Eighty-three (74%) of the students in the e-module group reported completing all six e-modules. There were 20 students (18%) who reviewed at least some of four or more modules and only nine (8%) who reported reviewing less than half of the modules.

### Short-term outcomes

**Fig. 1:**
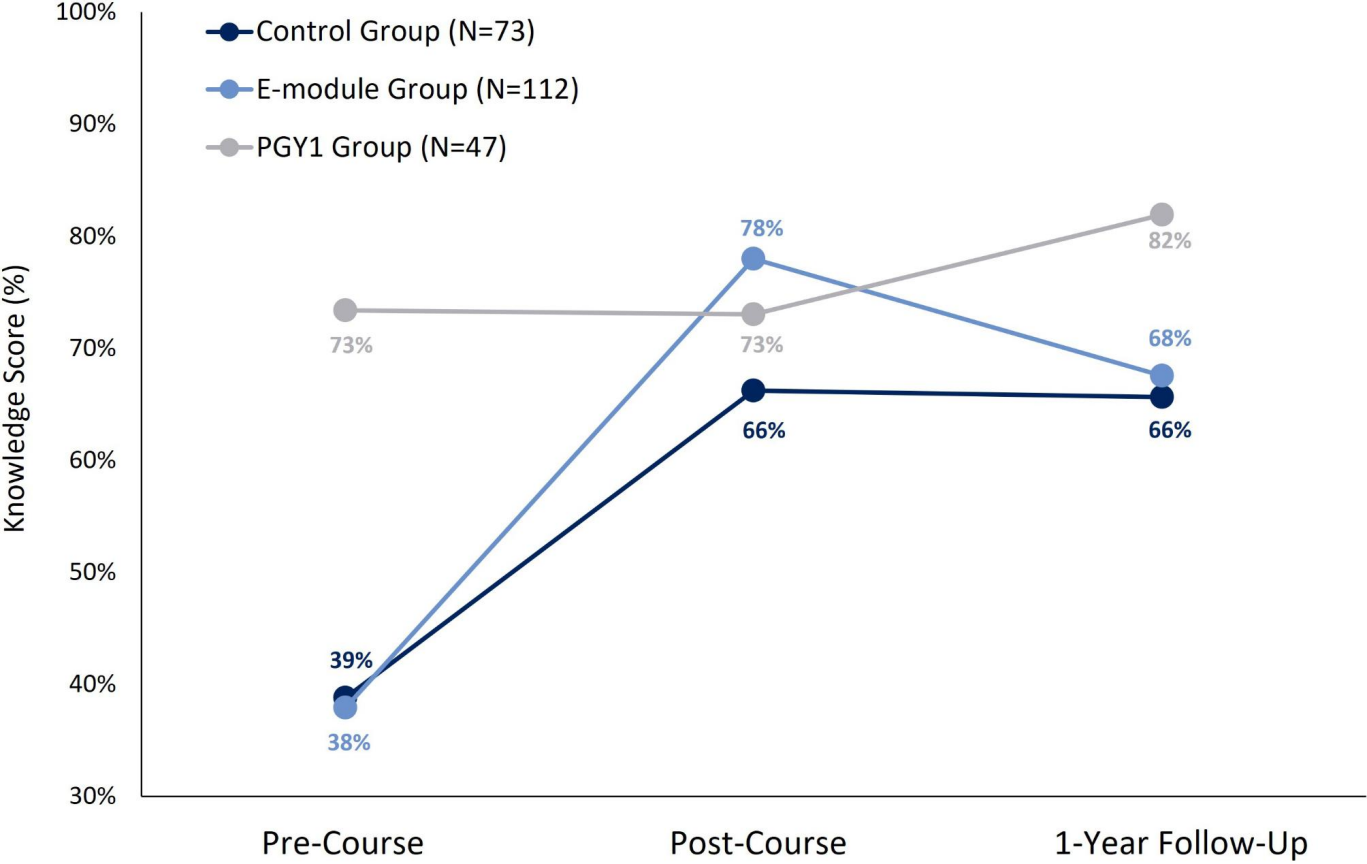
Mean percent knowledge scores in the control (black), e-module (blue) and PGY1 group (grey) at three time-points: pre-course, post-course and at 1-year follow-up. The e-module group performed better than the control group only at the post-course time-point (*p* = 0.003). Both medical student groups performed worse than the PGY1 group at the pre-course and 1-year follow-up (*p*-values = 0.003). At the 1-year follow-up, there was a smaller sample in the control (N = 50), e-module (N = 76), and PGY1 groups (N = 36)

Tables 2 and Fig. 1 shows the mean knowledge score for all three groups at each of the study time-points. Detailed statistical analysis results are presented in **Supplemental Material Appendix 2**. There was a significant difference between the three groups over time on knowledge score (group x time interaction: *F*(2,229) = 48.34, *p* < 0.001). Both the control and e-module groups’ knowledge scores improved over time (adjusted *p*-values < 0.003), however there was no change in PGY1s knowledge scores over time (adj *p* = 1.0). Pre-course knowledge scores did not differ between the control and e-module groups (39 vs. 38%, respectively, adj *p* = 1.0). However, the e-module group performed better than the control group on the post-course test (78% vs. 66%, respectively, adj *p* < 0.002). Compared to PGY1s, both the control and e-module group performed worse on the pre-test (adj *p*-values < 0.002) but the e-module group (adj *p* = 0.21) and the control group (adj *p* = 0.12) did not differ significantly from the PGY1 group at the post-course test.

**Table 2:** Mean (SD) knowledge percent scores (top) and mean/median (IQR) confidence scores for each group at the pre-course, post-course and 1-year follow-up time-points

Knowledge						
	Control Group (N = 73)		E-Module Group (N = 112)		PGY1 Group (N = 47)	
Pre-course score (%)	38.8 (18.6)		37.9 (20.4)		73.4 (16.2)	
Post-course score (%)	66.2 (19.8)		78.0 (16.9)		73.0 (18.2)	
1-year follow-up score (%)^†^	65.7 (21.9)		67.5 (17.6)		81.9 (13.4)	
**Confidence**						
	**Control Group (N = 66)**		**E-Module Group (N = 105)**		**PGY1 Group (N = 46)**	
	**Mean**	**Median (IQR)**	**Mean**	**Median (IQR)**	**Mean**	**Median (IQR**)
Pre-course score	17.8	17 (13.75–21.25)	17.8	18 (14.5–21.5)	27.7	27.5 (24.75-31)
Post-course score	35.0	35 (31.75-39)	34.6	35 (32–38)	29.9	30 (27–32)
1-year follow-up score^†^	32.8	32 (30.75–37.25)	33.6	33 (30.5–37.5)	31.3	32 (30–33)

^†^1-year follow-up had smaller sample sizes for knowledge data: Control (N = 50), E-Module (N = 76), PGY1 (N = 36); and confidence data: Control (N = 46), E-Module (N = 69), PGY1 (N = 35)

**Fig. 2:**
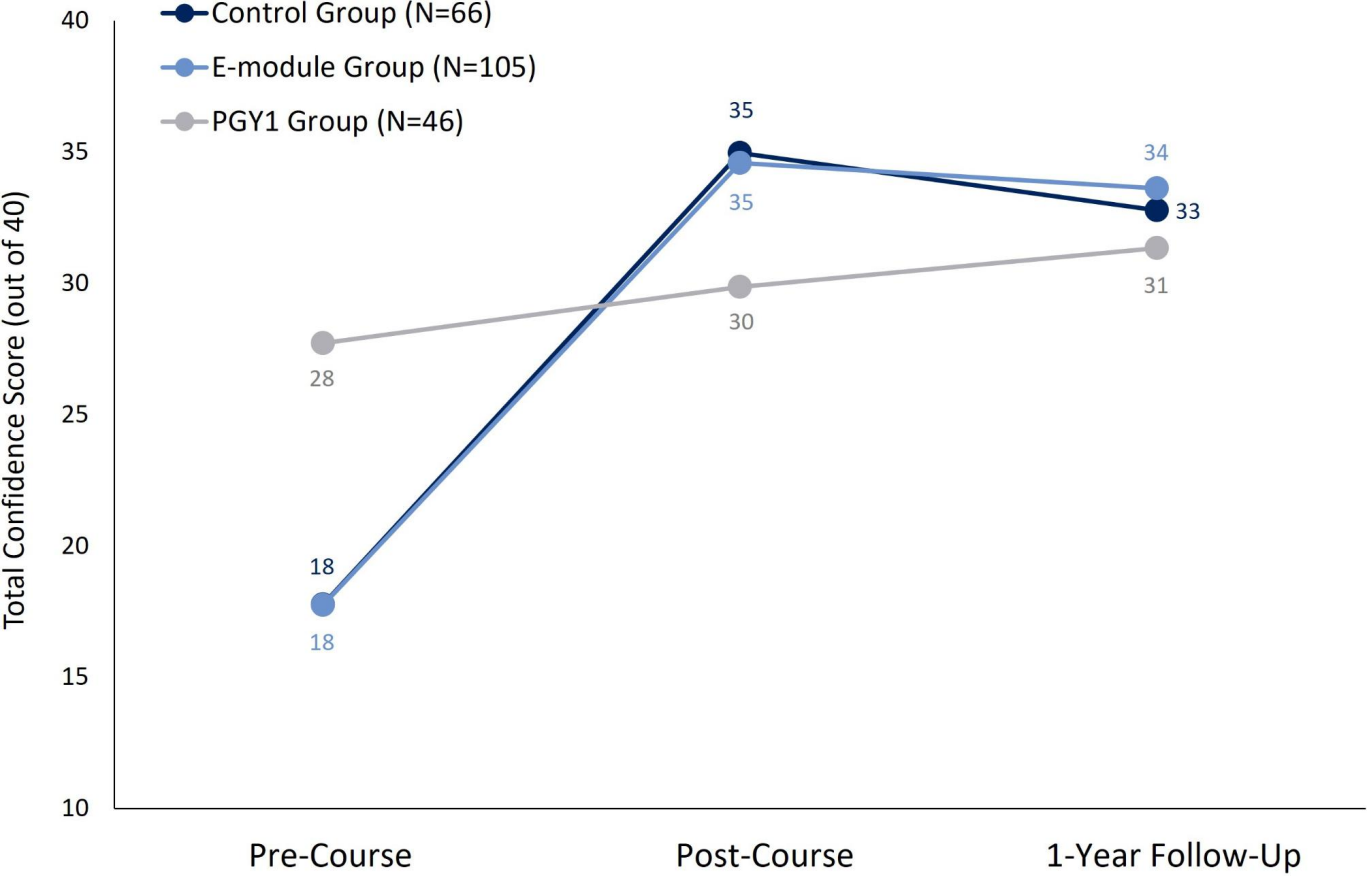
Mean confidence scores in the control (black), e-module (blue) and PGY1 group (grey) at three time-points: pre-course, post-course and at 1-year follow-up. Both medical student groups had lower confidence than the PGY1 group at the pre-course time-point (*p* = 0.003), but higher confidence at the post-course time-point (*p*-values = 0.003). The e-module group continued to have higher confidence compared to the PGY1 group at the 1-year follow-up (*p* = 0.03). There was a smaller sample at the 1-year follow-up in the control (N = 46), e-module (N = 69), and PGY1 groups (N = 35)

For the pre- and post-course confidence scores, 7 students in control group, 7 students in the e-module group, and 1 PGY1 resident reported “not sure” for at least half of the items, so they were removed from the analysis. Tables 2**and** Fig. 2 shows the mean confidence score for all three groups at each of the study time-points. At the pre-course test, the control and e-module group did not differ on their confidence score (adj *p* = 1.0), however both groups had significantly lower confidence scores than the PGY1 group (adj *p*-values < 0.003). At the post-course test, both the control and the e-module group had significantly higher confidence scores than the PGY 1 group (adj *p*-values < 0.003), but they did not differ from one another (adj *p* = 1.0). All three groups had a significant increase in scores from the pre- to post-course test (all *p-values* < 0.00).

**Fig. 3:**
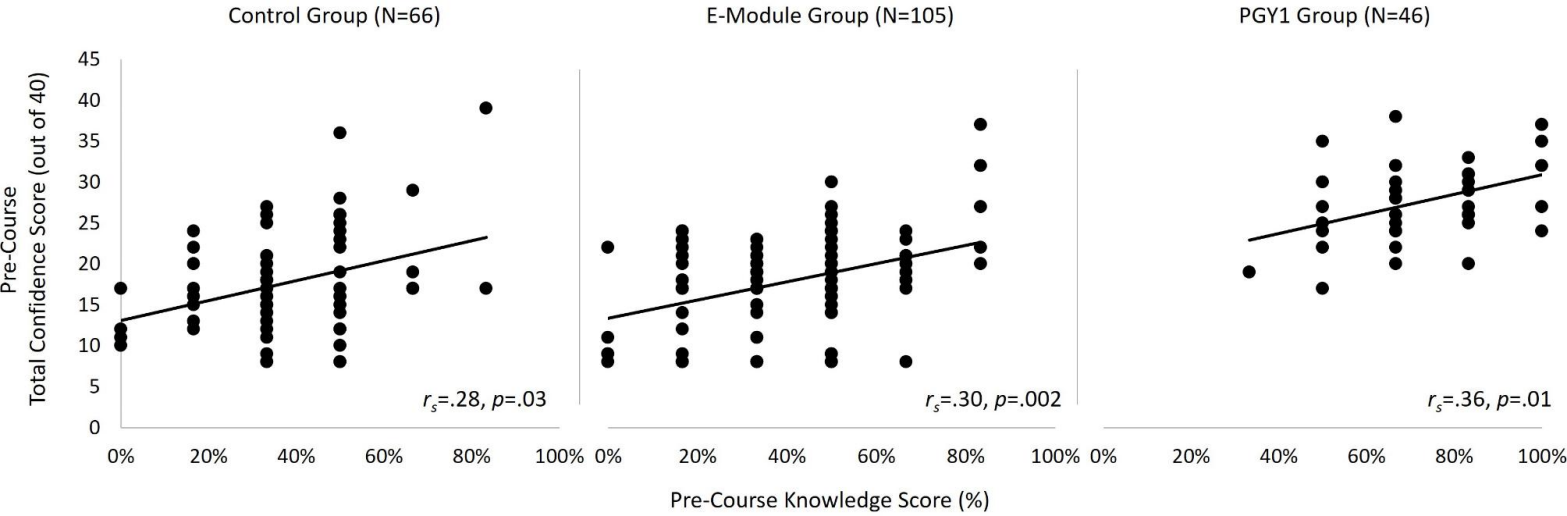
Scatterplots depicting the relationship between percent knowledge score and confidence score pre-course time-point for each group

There was a significant correlation between pre-course knowledge and confidence scores in the control (*r*_*s*_=0.28, *p* = 0.025), e-module (*r*_*s*_=0.30, *p* = 0.002), and PGY1 group (*r*_*s*_=0.36, *p* = 0.013; Fig. 3), however at the post-course test the only correlation that remained significant was for the PGY1 group (*r*_*s*_=0.60, *p* < 0.001).

### Long-term outcomes

There was a subsample of participants that completed the 1-year follow-up, which included 50 students in the control group (68%), 76 students in the e-module group (68%), and 36 PGY1 residents (77%). Detailed statistical analysis results are presented in **Supplemental Material Appendix 3**. To assure that this was a representative sub-sample, we compared post-test knowledge scores of students who did and did not participate in the 1-year follow-up within each group. There was no significant difference in performance between these groups (all *p*-values > 0.2).

There was a significant difference between the three groups over time on knowledge score (group x time interaction: *F*(4,318) = 15.52, *p* < 0.001; Tables 2**and** Fig. 1). The e-module groups’ knowledge scores decreased between the post-course test and 1-year follow-up (adj *p* = 0.004). Both the control and PGY1 group’s knowledge scores did not change (adj *p* = 1.0 and *p* = 0.14, respectively). One-year follow-up knowledge scores did not differ between the control and e-module groups (66% vs. 68%, respectively, adj *p* = 1.0), but both groups scored significantly lower than the PGY1 group (82%, adj *p*-values < 0.003).

At the 1-year follow-up, the control group did not differ from the e-module group (adj *p* = 1.0) or the PGY1 group (adj *p* = 0.26) on confidence scores **(**Tables 2**and** Fig. 2**).** However, the e-module group had significantly higher confidence scores than the PGY1 group (adj *p* = 0.03). The control group had a significant decrease in confidence scores between the post-course test and follow-up (adj *p* < 0.002), but there was no significant change in the e-module (adj *p* = 0.06) or the PGY1 group (adj *p* = 0.45).

There were no significant correlations between knowledge and confidence scores in the three groups at the 1-year follow-up (control: *r*_*s*_=0.28, *p* = 0.05; e-module: *r*_*s*_=0.22, *p* = 0.055; resident group: *r*_*s*_=0.29, *p* = 0.08; Fig. 3).

### Medical student resources used for learning ECG

There were three categories of resources that students reported using: course materials, textbooks and online. Course materials included PowerPoint materials used for small and large group sessions, a cardiology primer created by the CPR course director, and advanced cardiac life support (ACLS) training that took place at the start of the clerkship year. Textbooks that were often referenced included “Pathophysiology of Heart Disease” and “Rapid Interpretation of EKG’s.” Online resources that were utilized included https://boardsbeyond.com/, https://litfl.com/ecg-library/, https://www.youtube.com/, and https://www.healio.com/.

**Fig. 4:**
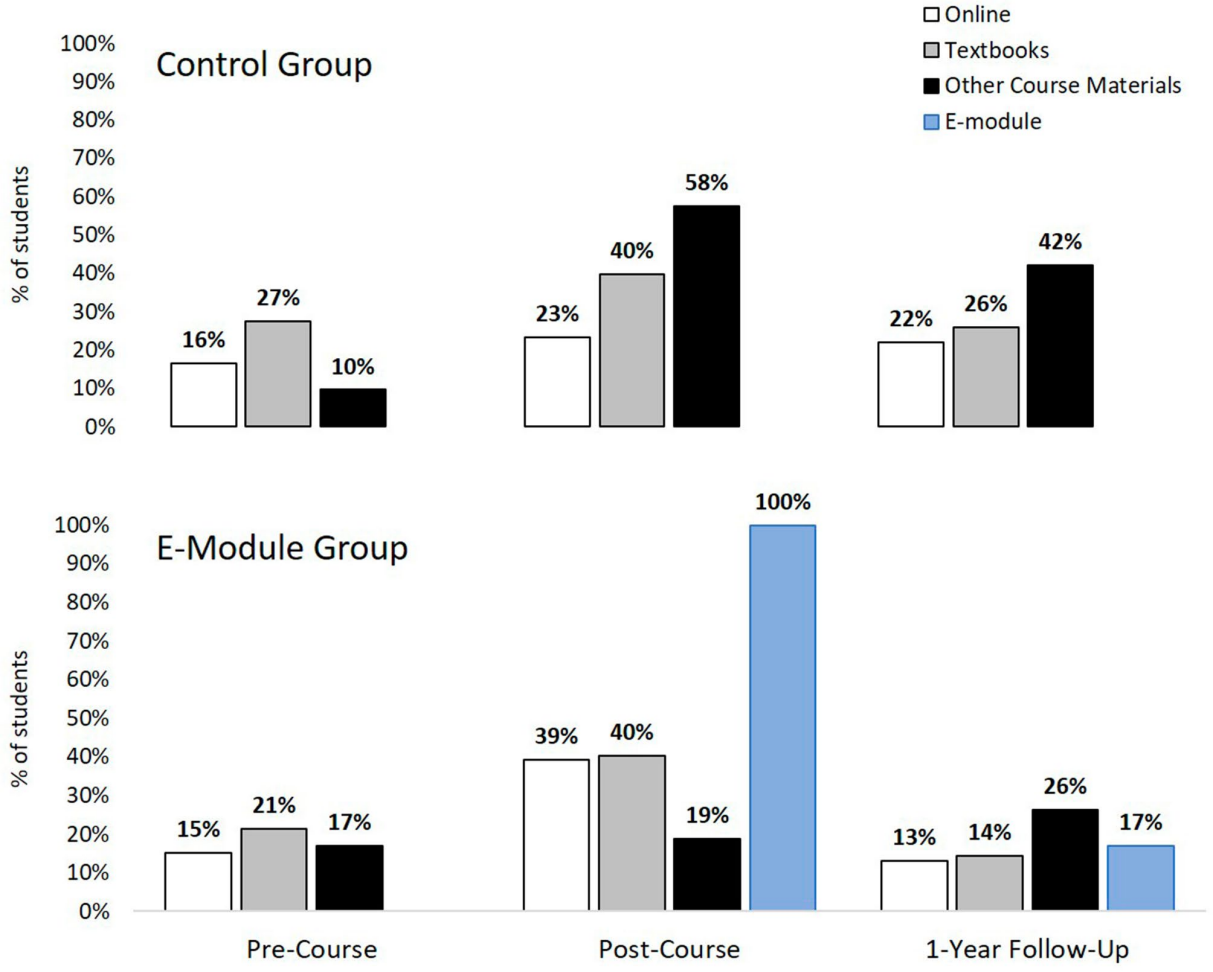
Percent of students who reported using various resources to learn ECG interpretation at each time-point in the control (top) and e-module groups (bottom). At the post-course time-point, the control group used other course materials more often than the e-module group (p < 0.001) and the e-module group used online resources more often than the control group (p = 0.02)

Figure 4 shows what type of resources students in each group used throughout the study to learn how to interpret ECGs. At the beginning of the CPR course, 32 students (44%) in the control group and 54 (48%) in the e-module group reported using at least one resource to learn ECG interpretation (*χ*^*2*^(1) = 0.34, *p* = 0.56). There was no significant difference between the two groups on what types of resources the used (all *p*-values > 0.05). Both groups most often reported using textbooks and online resources, as well as course materials (e.g., lectures and cardiology primer).

At the end of the CPR course, significantly more students in the control group (N = 63, 86%) reported using at least one resource to learn ECG interpretation compared to the e-module group (N = 82, 73%; *χ*^*2*^(1) = 4.47, *p* = 0.04). The control group reported using course materials significantly more than the e-module group (*χ*^*2*^(1) = 29.6, *p* < 0.001), whereas the e-module group used online resources significantly more than the control group (*χ*^*2*^(1) = 5.1, *p* = 0.02). The groups did not differ on their use of textbooks (*χ*^*2*^(1) = 0.0, *p* = 0.95), however students now reported using the book “First Aid for the USMLE Step 1” in addition to other traditional cardiology textbooks.

At the 1-year follow-up, 33 students (66%) in the control group and 46 students (61%) in the e-module group reported still using at least one resource to learn ECG interpretation (*χ*^*2*^(1) = 0.39, *p* = 0.53). There was no significant difference between the two groups on what types of resources they used (all *p*-values > 0.05). Both groups continued to rely mostly on course materials, however students described their clerkship experience or ACLS training when referring to course materials. Nineteen (25%) of the e-module students reported using the e-module at the follow-up.

## Discussion

We found that MS1 students using an asynchronous, interactive e-module to learn ECG interpretation resulted in better short-term acquisition of knowledge than providing the same material through a didactic lecture. The use of e-learning to teach ECG interpretation to students in the preclinical phase of the curriculum has not been extensively reported in the literature, although one study did find that e-learning was not effective compared to near-peer teaching [39]. Our findings are comparable to what is reported when e-learning is utilized during the clinical years, namely that e-learning is an effective way to teach ECG interpretation [13, 31–33, 36]. A blended learning model is considered ideal [13, 33] but we’ve shown that asynchronous access to an e-module can be successful when used independently. There is a great deal of variability in the methods used to teach ECG interpretation through an electronic format, making it difficult to make direct comparisons among the literature [41].

Studies typically show a loss of ECG interpretation skill over time [13, 31], which is comparable to our findings. This is likely due to the student’s lack of engagement with the material over time, which is heavily reliant on a students’ motivation for learning [4, 23]. The American College of Cardiology and the American Heart Association recommend a minimum of 500 ECG are necessary for doctors trained in internal medicine or cardiology to become competent in ECG interpretation, with continued practice of at least 100 per year to retain those skills [42]. Only 40% of internal medicine clerkship directors indicated that medical students are expected to interpret a minimum of 9–10 ECGs during their clerkship [3], which is an expectation that is far below what is expected for competence. In our study, by the 1-year follow-up students were about one month into their clinical clerkship year so they had limited exposure to ECGs in practice. Few students also reporting continued use of the e-module which is likely why their performance declined in the year following the course. For the PGY1 trainees, they had a non-significant increase in their knowledge scores between the post-test and 1-year follow-up. This is likely due to their on-the-job exposure to ECG interpretation as a resident, which included time in the Cardiac Acute Care Unit (the primary cardiology inpatient service).

Regardless of which method of teaching ECG interpretation, students’ confidence significantly increased by the end of the cardiology course and remained high even one year later. This is consistent with other studies that show higher confidence regardless of the intervention [13, 34, 36, 39, 43]. Our students had such high confidence levels that they exceeded our PGY1 residents’ confidence scores. Confidence, however, does not relate to skill level as our students’ confidence and knowledge scores did not correlate beyond the pre-course time-point. Other studies have confirmed the disconnect between confidence in skills such as ECG interpretation [13] and other areas of competence [44, 45].

Generation Z or Millennial learners typically prefer using online resources and videos to deliver content [26, 27], yet several studies have shown that medical students still use traditional resources in conjunction with online material [46, 47]. In line with this, we found that many students reported using textbooks and course materials for learning ECG interpretation, both before the formal start of the course and one year after the course was completed. Some of the resources were aimed at studying for the USMLE Step 1 exam (e.g., “First Aid for the USMLE Step 1”) which is a significant concern for students once they start their clinical year [48]. Online resources were used, but to a lesser extent than other types of resources. Students rarely described using websites such as YouTube or Google, which is a positive sign because there is concern that students may be using resources that are unreliable [49, 50].

In recent years there has been a push towards introducing clinical experiences earlier on in the medical school curriculum. In doing so, the traditional 2 + 2 format has evolved into various formulations that typically include the reduction of time spent learning the foundational basic sciences [51–53]. There is also concern that the recent move to make the USMLE Step 1 exam pass/fail will reduce incentive for students to learn the basic sciences [54, 55]. ECG interpretation is a clinical skill that requires a thorough understanding of the underlying physiology of the heart, representing the ideal example of the interdependence of the basic and clinical sciences. Taking advantage of online resources to provide the foundational basic science to students can assure that students are gaining the knowledge they need early enough in the curriculum so they can practice applying these skills in their expanding clinical experiences. This can also allow educators to save synchronous curriculum time for application exercises.

There are some limitations to our study that are important to note. The study includes data from a single institution that had implemented a leaner-centered model emphasizing self-regulated learning. Therefore, our students may have been exceptionally comfortable learning independently because that is an expectation of the curriculum overall. The survey used to determine confidence and ECG interpretation skills was not a validated survey. We had a low response rate for the control group’s pre-post survey data. Regardless of this, between 68 and 77% of students and residents completed the follow-up survey, which represents a good portion of the study sample. There appears to be some sample bias due to the fact that participants who were not included in the study (i.e., did not complete the post-test) performed worse on the pre-test than those who were included in our study. Thus, this sample may not be fully representative of all medical students. Unfortunately, many of the students in the e-module group did not continue to use this resource to practice ECG interpretation in the follow-up year which limited our ability to assess its effectiveness for long-term retention. Finally, because the e-module was created locally and housed behind the university’s firewall we are unable to distribute it widely thus limiting its use for the medical education community.

## Conclusion

We showed that the use of an asynchronous, e-module is an effective way to teach ECG interpretation in first-year medical students. With significant reductions in preclinical curricular time, educators can consider the use of e-modules to deliver basic concepts, which can be reinforced and practiced synchronously during small group learning sessions. Future studies to better understand how continued practice could impact long-term retention of ECG skills would make a substantial contribution to this literature.

## Electronic supplementary material

Below is the link to the electronic supplementary material.

Supplementary Material

## Data Availability

The datasets used and/or analyzed during the current study are available from the corresponding author on reasonable request.

## List of abbreviations

ACLS: advanced cardiac life support
Adj: adjusted
ANOVA: analysis of variance
CRP: Cardio-Pulmonary-Renal course
ECG: electrocardiogram
IQR: interquartile range
LEARN: Learning focused, Experiential, Adaptive, Rigorous and Novel curriculum
MS1: first-year medical students
MS2: second-year medical students
MS3: third-year medical students
PGY1: First year residents
RSOM: Renaissance School of Medicine at Stony Brook University
SD: standard deviation
SDL: self-directed learning
