# Supplementary material for: Comparing the effectiveness of asynchronous e-modules and didactic lectures to teach electrocardiogram interpretation to first year US medical students
Source: BMC Med Educ. 2023 May 22;23:360. doi: 10.1186/s12909-023-04338-6 (PMC10201768; doi:10.1186/s12909-023-04338-6)
Supplement: Supplementary file 1 — Supplementary Material [file 12909_2023_4338_MOESM1_ESM.docx]

Supplemental Material Appendix 1

Screenshots of the e-module show the flow of the individual modules to the left (A) and the main slide deck presenting the material. Animated handwritten text was used to pace the material and coincide with the audio track (B). At set intervals, which are tagged along the module’s timeline (C), questions would pop-up related to the content that was previously covered. Students could track their performance on past questions and see how many more questions were left within each module (D). In-module questions were followed by detailed explanations and students could choose to review the content, retry answering the question, or continue (F). Students were able to increase or decrease the speed of the module based on their learning style and select closed captioning (E). At the end of each module there was a quiz to reinforce key concepts and apply what was learned to clinical scenarios (G). For questions that required direct analysis of an ECG image, calipers were available to assist with calculating the duration of the interval (H). An ECG cheat sheet was available for reference with definitions of key terms (I).


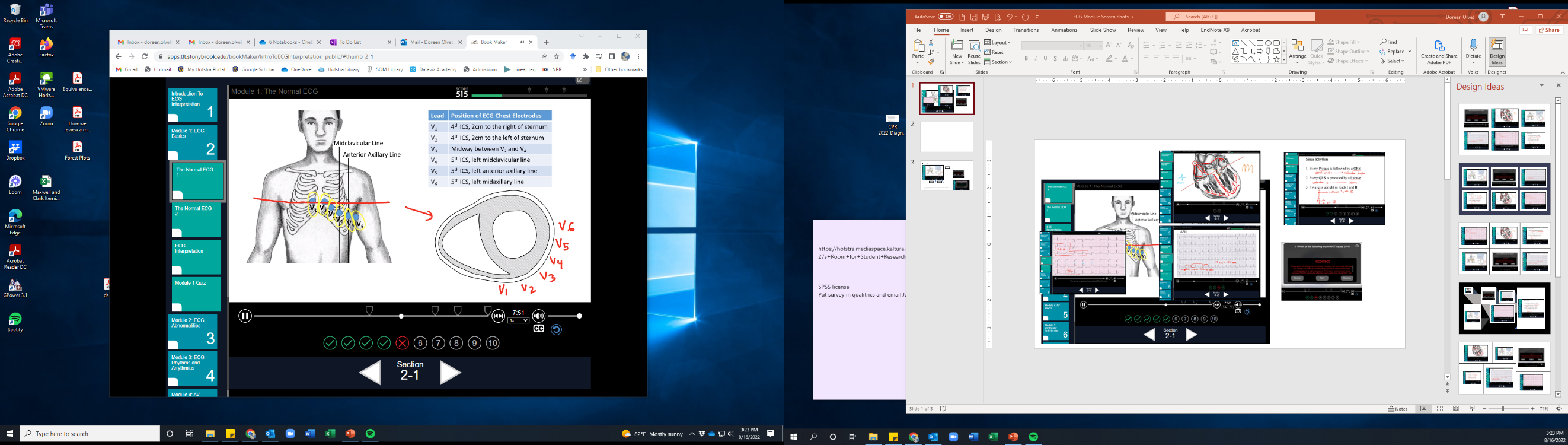


**B**

**C**

**D**

**E**

1. Tracking modules
2. Animated handwritten text
3. In-module questions
4. Tracking performance on in-module questions
5. Speed & accommodations
6. Immediate performance feedback
7. End of module quiz & feedback
8. Virtual calipers
9. ECG cheat sheet

**A**


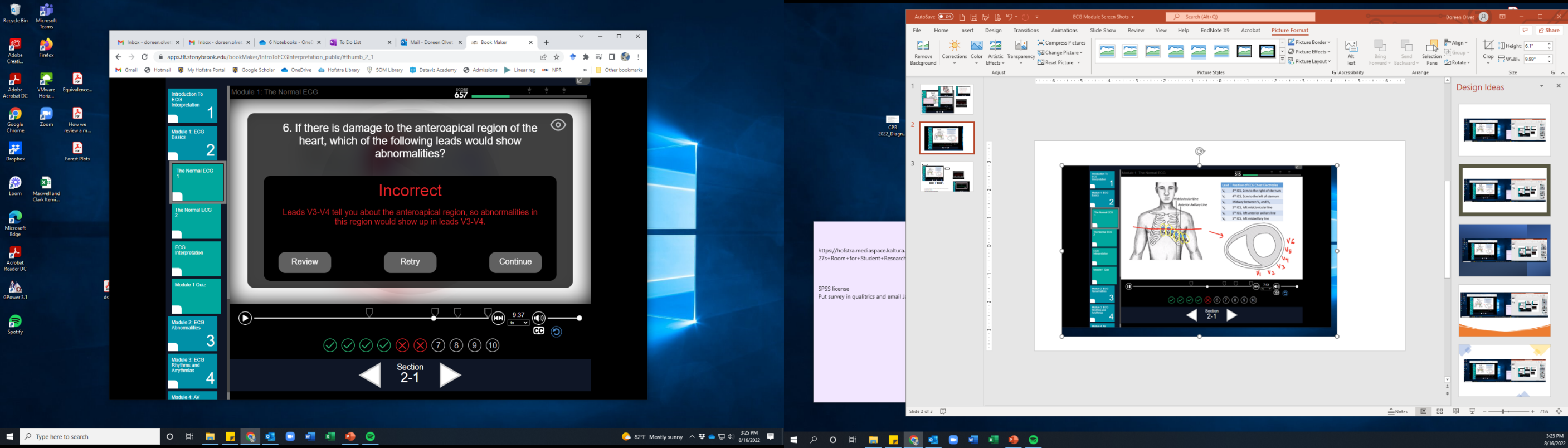


**F**


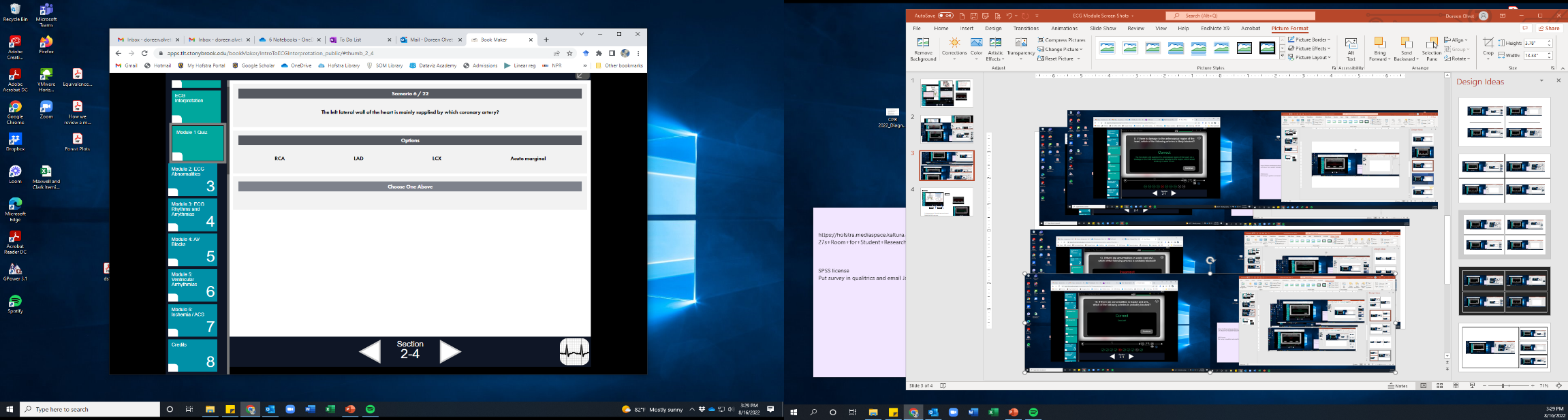

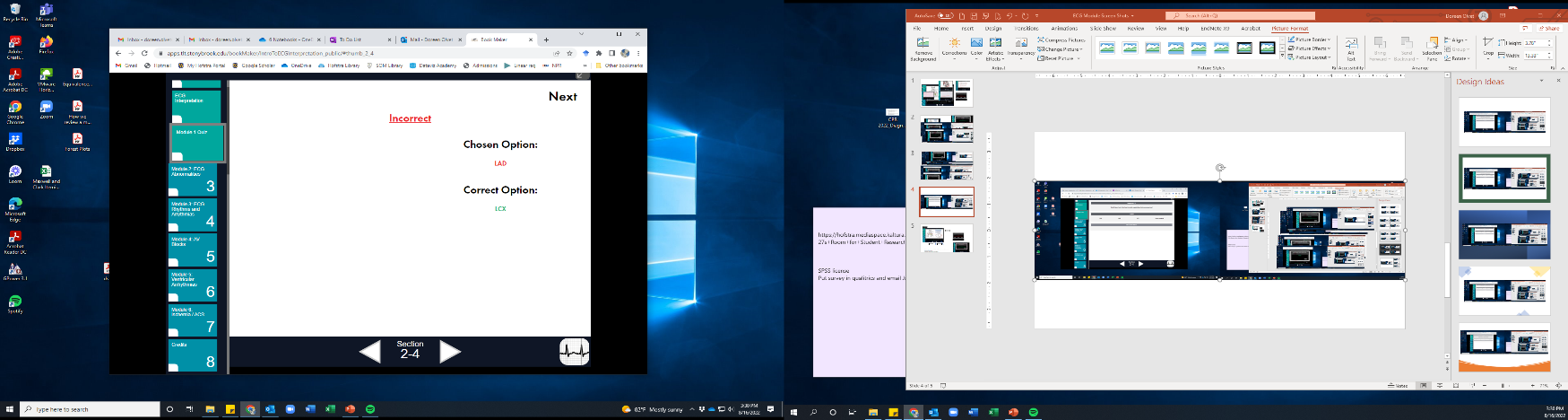


**G**


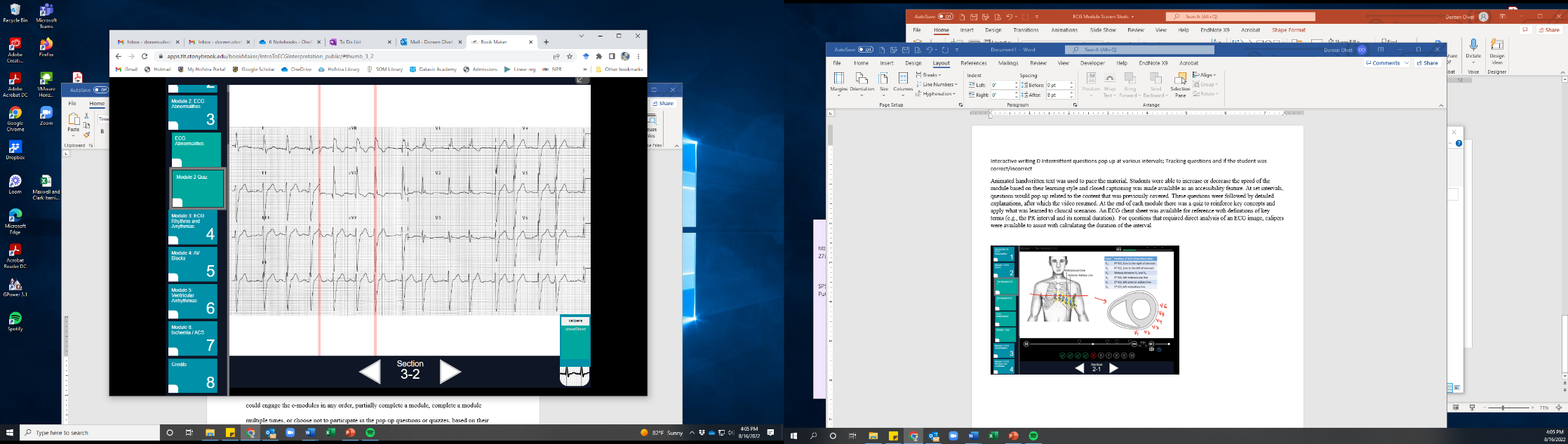


**H**


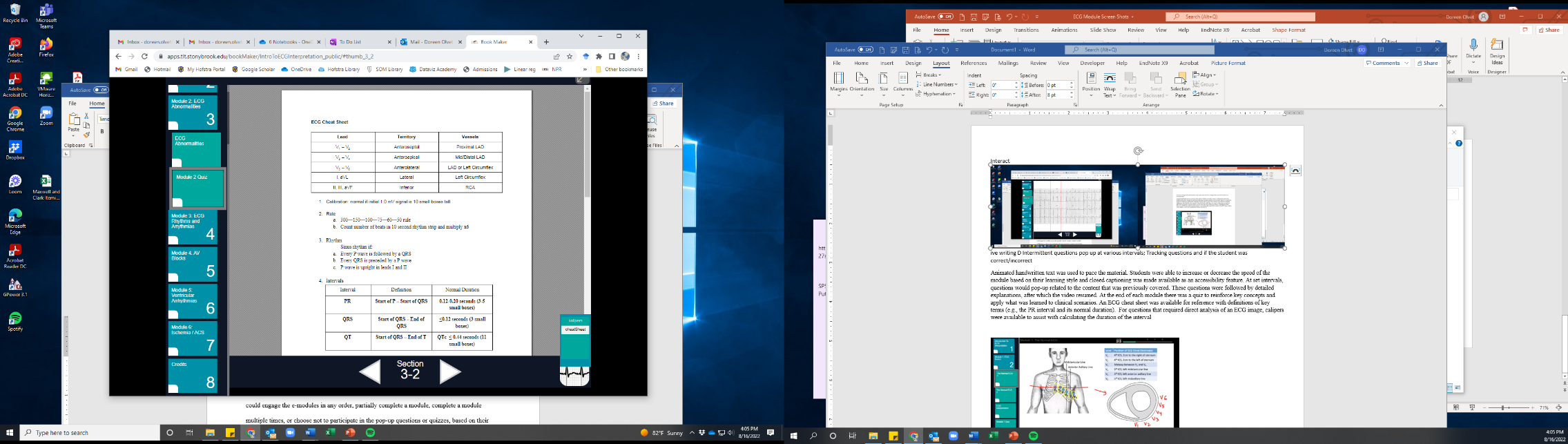


**I**

Supplemental Material Appendix 2

Detailed results for the statistical analysis of short-term outcomes comparing the three groups at the pre-course and post-course time-points. For knowledge scores, results from the mixed ANOVA are presented, as well as the post-hoc tests using a paired t-test for time (within-subjects) and a Student’s t-test for group (between-subjects) at each time-point. For the confidence scores, a Wilcoxon Signed Rank test was performed for time (within-subjects) and a Mann-Whitney U test for group (between-subjects). P-values are adjusted for multiple comparisons.

| Knowledge | | |
| --- | --- | --- |
| Mixed ANOVA Results | | |
| Time | *F*(1,229)=183.39, *p*<0.001 | |
| Group | *F*(2,229)=30.66, *p*<0.001 | |
| Group x Time | *F*(2,229)=48.34, *p*<0.001 | |
| Post-hoc tests | | |
| Time (within-subjects) | Control Group (N=73) | Pre vs. Post: *t*(72)=-8.77, *p*<0.003 |
|  | E-Module Group (N=112) | Pre vs. Post: *t*(111)=-17.78, *p*<0.003 |
|  | PGY1 Group (N=47) | Pre vs. Post: *t*(46)=0.14, *p*=1.0 |
| Group (between-subjects) | Control vs. E-Module | Pre: *t*(183)=0.29, *p*=1.0  Post: *t*(183)=-4.32, *p*<0.002 |
|  | Control vs. PGY1 | Pre: *t*(118)=-10.44, *p*<0.002  Post: *t*(118)=-1.90, *p*=0.12 |
|  | E-Module vs. PGY1 | Pre: *t*(157)=-10.59 *p*<0.002  Post: *t*(157)=1.64, *p*=0.21 |
| Confidence | | |
| Time (within-subjects) | Control Group (N=66) | Pre vs. Post: *Z*=-7.07, *p*<0.001 |
|  | E-Module Group (N=105) | Pre vs. Post: *Z*=-8.72, *p*<0.001 |
|  | PGY1 Group (N=46) | Pre vs. Post: Z=-3.45, *p*=0.001 |
| Group (between-subjects) | Control vs. E-Module | Pre: *U*=3226.5, *p*=1.0  Post: *U*=3316.5, *p*=1.0 |
|  | Control vs. PGY1 | Pre: *U*=316.5, *p*<0.003  Post: *U*=607.5, *p*<0.003 |
|  | E-Module vs. PGY1 | Pre: *U*=399, *p*<0.003  Post: *U*=958, *p*<0.003 |

Supplemental Material Appendix 3

Detailed results for the statistical analysis of long-term outcomes comparing the three groups at the pre-course, post-course and 1-year follow-up time-points. For knowledge scores, a mixed ANOVA and post-hoc t-tests were performed using a paired t-test for time (within-subjects) and a Student’s t-test for group (between-subjects) at each time-point. For the confidence scores, a Wilcoxon Signed Rank test was performed for time (within-subjects) and a Mann-Whitney U test for group (between-subjects). P-values are adjusted for multiple comparisons.

| Knowledge | | |
| --- | --- | --- |
| Mixed ANOVA Results | | |
| Time | *F*(2,318)=68.51, *p*<0.001 | |
| Group | *F*(2,159)=31.40, *p*<0.001 | |
| Group x Time | *F*(4,318)=15.52, *p*<0.001 | |
| Post-hoc tests | | |
| Time (within-subjects) | Control Group (N=50) | Pre vs. Post: *t*(49)=-6.55, *p*<0.002  Post vs. 1YR: *t*(49)=-0.47, *p*=1.0 |
|  | E-Module Group (N=76) | Pre vs. Post: *t*(75)=-12.38, *p*<0.002  Post vs. 1YR: *t*(75)=3.23 *p*=0.004 |
|  | PGY1 Group (N=36) | Pre vs. Post: *t*(35)=0, *p*=1.0  Post vs. 1YR: *t*(35)=-1.87, *p*=0.14 |
| Group (between-  subjects) | Control vs. E-Module | Pre: *t*(124)=0.40, *p*=1.0  Post: *t*(124)=-3.87, *p*<0.003  1YR: *t*(124)=-5.30, *p*=1.0 |
|  | Control vs. PGY1 | Pre: *t*(84)=-9.61, *p*<0.003  Post: *t*(84)=-2.97, *p*=0.01  1YR: *t*(84)=-3.95, *p*<0.003 |
|  | E-Module vs. PGY1 | Pre: *t*(110)=-8.57, *p*<0.003  Post: *t*(110)=0.42, *p*=1.0  1YR: *t*(110)=-4.34, *p*<0.003 |
| Confidence | | |
| Time (within-subjects) | Control Group (N=46) | Pre vs. Post: *Z*=-5.91, *p*<0.002  Post vs. 1YR: *Z*=-4.16, *p*<0.002 |
|  | E-Module Group (N=69) | Pre vs. Post: *Z*=-7.00, *p*<0.002  Post vs. 1YR: *Z*=-2.17, *p*=0.06 |
|  | PGY1 Group (N=35) | Pre vs. Post: Z=-3.68, *p*=0.002  Post vs. 1YR: *Z*=-1.21, *p*=0.45 |
| Group (between-  subjects) | Control vs. E-Module | Pre: *U*=1407, *p*=0.91  Post: *U*=1361 *p*=0.58  1YR: *U*=1488.5, *p*=1.0 |
|  | Control vs. PGY1 | Pre: *U*=182, *p*<0.003  Post: *U*=303, *p*<0.003  1YR: *U*=626.5 *p*=0.26 |
|  | E-Module vs. PGY1 | Pre: *U*=231.5 *p*<0.003  Post: *U*=525, *p*<0.003  1YR: *U*=835, *p*=0.03 |
